# The Need for Improved Recruitment to Neurosurgery Training: A Systematic Review of Enrollment Strategies

**DOI:** 10.7759/cureus.26212

**Published:** 2022-06-22

**Authors:** Chukwuyem Ekhator, Ramin Rak

**Affiliations:** 1 Neuro-oncology, New York Institute of Technology, College of Osteopathic Medicine, Old Westbury, USA; 2 Neurosurgery, Rockville Center, New York, USA

**Keywords:** surgery, enrollment, education, neurology, neurosurgery

## Abstract

Neurosurgery is one of the cornerstones corresponding to a large scope of clinical pathologies and is a highly-regarded surgical specialty. However, there has been a decline in recruits into the neurosurgical residency due to many factors derailing the interest of medical students with an ambition to become neurosurgeons. Some of these issues encompass little or lack of early exposure to neurosurgery, lack of quality mentorship programs, and institutional curriculum entailing prolonged periods of training and study in neurosurgery. Therefore, this systematic review and meta-analysis aim to establish some strategic methodologies for increasing the recruitment to neurological surgery. Neurosurgery is an interestingly exciting specialty that integrates cutting-edge technology allowing for diversified subspecialization with an exceptional degree of variety. Nevertheless, several factors such as the duration of the required training, the kind of lifestyle, lack of early exposure to neurosurgery, and lack of mentors to a vast of medical students across the globe have curtailed the recruitment to neurological surgery. Despite an increased number of female representations in medical surgery, there has been a reported increase in students matching into neurosurgery, although the number is relatively below the expectation due to the factors highlighted earlier. As a result, many studies and surveys have been conducted to identify ways of improving neurosurgical recruitment.

Five electronic databases, including PubMed, Science Direct, Cumulative Index to Nursing and Allied Health Literature (CINAHL), Google Scholar, MEDLINE, and Cochrane Library, were searched to provide pertinent information to the topic of study in strict compliance with the PRISMA (Preferred Reporting Items for Systematic Review and Meta-analysis) guidelines. Meta-analysis was then conducted on the included studies to determine their correlations based on the individual outcomes of each study. A total of 2,134 search results were obtained, screened, and reviewed against the exclusion and inclusion criteria to remain with 12 included studies detailing improving the recruitment to neurosurgical residency. The 12 studies were retrieved for their study characteristics based on the PICO (predetermined patient, intervention, control, and outcome) standards. Most of the studies were surveys (n = 8), retrospective and prospective studies (n = 2), and pilot and multifocal studies making up for the rest (n = 2). Several neurosurgery aspects need consideration to improve the recruitment of medical students to neurosurgical fields. Medical institutions, specialists, and other stakeholders should consider reconstructing the neurosurgical curriculum to ease the prolonged study time as well as to create and encourage structural programs aimed at acquainting medical students in neurosurgery and involving the students in conducting other research projects. In addition, mentorship programs and early exposure of medical students to neurological surgery play a key role in influencing the medical students' interest in choosing neurosurgical career paths.

## Introduction and background

Neurosurgery is an interestingly exciting and dynamic specialty that heavily embraces the integration of cutting-edge technology, allowing for diversified subspecialization with an exceptional degree of variety. Owing to this, the expectations are that neurological surgery should make up the most popular career path for many medical school graduates. However, this is not the case as the training required for neurological surgery poses a run-through of perseverance training. For this reason, there has been a significant decline in the number of individuals in the neurological surgery application in the recent past. Although it is difficult to correctly ascertain the accurate factors and reasons behind a declined neurosurgery resident applicant, there are a few reasons that may likely explain the overall decline and shortages in the number of neurosurgical residents. The decreased number of neurosurgery residency applicants can be articulated to a number of factors, which encompass the duration of the required training, the kind of lifestyle, lack of early exposure to neurosurgery, and lack of mentors for a vast of medical students across the globe [1-3]. Nevertheless, neuroscience is reportedly among the most common majors that undergraduate premedical students have over the years shown interest in furthering their careers in neurological illnesses and disease [4,5].

Although with time, medical students tend to shift their interests to another medical specialty in the early stages of their medical schooling. The tendency can be attributed to factors mentioned earlier, including insufficient and late exposure and experience with clinical neurological surgery. Neurological exposure is always reduced because medical students have to go through several rotations, scheduling difficulties, and being curtailed by the elevated emphasis on extra-classroom (outdoor) academic activities [6,7]. According to a survey conducted on medical school deans in the United States, 59% of the correspondents suggest that neurological surgery should not be a mandatory requirement for a rotation. In contrast, 33% of the deans only offered neurological surgery rotations before the medical students reached their fourth year of study [6]. Besides, suppose the schools were to offer clinical neurological surgery rotations in the third- or fourth-year level. In that case, a considerably large number of the students often decide on their specialty before such critical clinical years of their medical schooling [8]. The insufficiently limited exposure to the practical aspects of neurological surgery in the pre-clerkship period culminates in a missed chance and a great opportunity to change medical students' interests and influence their choice positively before making their final decision on the career path they would take [9]. This is because positive experiences from exposure to the actual and practical neurosurgery during the surgical clerkship would play a pivotal role in enhancing and influencing the students' preferences regarding their surgical career path [10,11]. In addition, exposure will also increase their interest in surgical medical schooling, which is a form of motivation, with earlier exposure stimulating the specific interest in neurosurgery.

Furthermore, the downtrend in the applicants of neurosurgery residents is exacerbated by less female representation in medical surgery. It has been reported that female students are less likely to engage in surgical training compared to their male counterparts [12]. This trend can be articulated in the fact that neurosurgery, like other forms of surgical training, requires mastering both science and art, heavily on long working hours to get acquainted and gain the much-required operative skills [13]. For this reason, working reforms have been adjusted in various regions across the continent to minimize the time demand for neurosurgery, also owing to the fact that surgical training is considered the most time-intensive and -consuming of all training specialties [12]. As a result, a number of medical students may likely opt for a less demanding and less time specialty [1]. In addition, some of these factors provide an explanation for why medical students prefer a more balanced work-life. For this reason, there is a trend among the medical students to pursue specialties presenting with more life-friendly kind of lifestyle and eventually end up in specialties such as ophthalmology, radiology, dermatology, and anesthesiology. Turning to such specialties serves to leverage the students from issues associated with neurosurgery, such as job dissatisfaction, depression, long periods of surgical training in the neurological residency, and anxiety disorders [14-16]. Therefore, due to the demanding nature of neurological surgery, many medical students (about 90%) would consider neurosurgery an astounding specialty but get reluctant to pursue neurological careers [17]. Similarly, there has been a consensus for increased female student representation in the medical school, which has to some extent exacerbate the decline of neurosurgery residence applicants since most female students are more likely to choose specialties with emphasis on lifestyle-friendly factors [18,19].

By 2003, the number of female student applicants to the surgical residency had significantly declined, comprising as low as 30% of the residency program. However, with the increased sensitization of equal representation of both genders, there has been a significant increase in the number of females registering for surgical training [20]. Despite having equal representation in the medical field, women still account for a small number of neurosurgery residents [21]. A white paper was published by the leadership of WINS (Women in Neurological Surgery) outlining some of the key issues that are common among female neurosurgery residents [18]. The report emphasized issues such as lack of mentorship over barriers to the kind of lifestyle they all want as the key barrier to surgical residency recruitment, especially during early medical school. This systematic review and meta-analysis examine the several ways that can be used to improve recruitment to neurosurgery. This objective can be realized by looking at the problems and issues hindering surgical residency application as highlighted by the research studies discussed above.

Materials and methods

Study Design

A multiprocess evaluation of improving the recruitment to neurological surgery was performed in the study design of this systematic review and meta-analysis. In so doing, some of the methodologies and interventions employed to improve the process of increasing the number of neurosurgical applicant residents were identified, gauged, and then reviewed. The studies exploring the neurological surgery recruitment of residents were obtained and separately categorized, analyzed, and evaluated to establish how to improve the process of recording and increasing the number of neurosurgeons. In addition, the context of this article also explores some of the challenges and issues that hinder medical graduates from choosing neurological surgery as their specialty.

Information Sources and Study Searches

A well-outlined search was conducted to navigate and explore a number of electronic databases for the primary article relevant to the topic of this article. Some of the medical-based electronic databases utilized in search of pertinent information to the issue of discussion include PubMed, MEDLINE, Cochrane Library, Science Direct, Google Scholar, and Cumulative Index to Nursing and Allied Health Literature (CINAHL). The advanced search tab contained in these databases was used to search for key terms and the entire topic heading of this systematic review and meta-analysis. To acquire more content, scope, and a vast pool of information, some references to the included studies were retrieved and then evaluated through them for relevance to improving the recruitment of medical students into neurosurgery.

Eligibility Criteria

Considerate inclusion and exclusion criteria were applied in this article to obtain only pertinent information from studies deemed key for this systematic review and meta-analysis. The studies considered for inclusion in this systematic review were evaluated against the inclusion criteria (Table 1).

**Table 1:** The inclusion and exclusion criteria of the included studies All the included studies were accessible via the internet sources explored (electronic databases). On the other hand, payments were done for articles requiring special permissions to access.

Inclusion criteria	Exclusion criteria
Studies published and available in the English language	Studies were done in other languages other than the English language
Studies that explored neurological surgery	Studies that explored improving recruitment to other forms of surgery other than neurosurgery
Primary studies that encompassed increasing the number of neurosurgery applicant residents	Other articles and case studies that reported on the neurosurgery recruitment of medical students such as newspaper articles, other systematic reviews, and case reports
Studies published between 2000 and 2022	

Search Strategy

**Figure 1:**
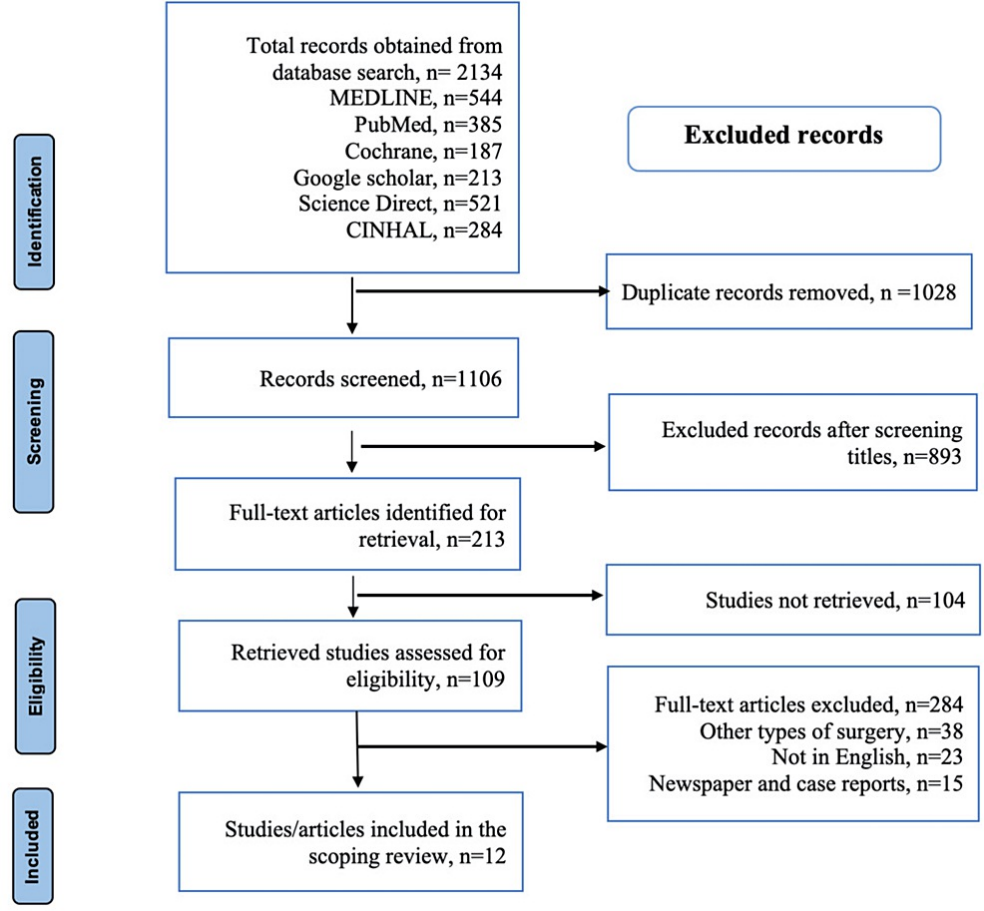
PRISMA table showing the flowchart diagram on the literature search and the process employed in search of information This figure summarizes the search strategy; the electronic databases explore the number of studies obtained from each one of them in search of pertinent information for this systematic review and meta-analysis. Additionally, the flowchart provides a procedural screening of articles based on inclusion and exclusion criteria.

In complete compliance with the PRISMA guidelines, a comprehensive search strategy was conducted through the aforementioned medical databases to determine how to improve the recruitment to neurological surgery. By employing key terms such as surgery, neurology, recruitment, neurological, and neurological surgery in the advanced tabs of the electronic databases, a total of 1,651 studies were identified from the first search. The second phase of the search encompassed some of the references of these studies, generating a total of 483 articles. Moreover, for effectiveness in searching for pertinent information, some searches considered the full title of the topic under study. Finally, the literature search and the outcomes of the screening method were then summarized in the PRISMA table, as illustrated in Figure 1.

Data Extraction and Analysis

Two independent reviewers were consulted and employed in analyzing and assessing the obtained studies to gauge their suitability for inclusion in this systematic review and meta-analysis. In addition, the researchers were also charged with looking for the possibilities of unscreened information and data on increasing the number of neurological surgery residents' entries and applicants. Therefore, the data extraction and study selection process strictly conform to the PICO (predetermined patient, intervention, control, and outcome) criterion. Thus, the reviewers could draw out key characteristics of the included studies encompassing study design, participants (size), authors, outcomes, and years of publications. The total average entry and matching of medical students into neurosurgery residencies were calculated after the various interventions were employed annually. Besides, the percentages of students' interest groups were also calculated based on the surveys administered to various participants based on Likert-scale queries. Additionally, percentage entry, mean, median, and standard deviations of the student entry into neurosurgical faculty were derived in other studies with various interventions for improvement.

## Review

Results

Study Characteristics

This study includes a total of 12 articles out of 2,134 studies, which were employed for inclusion after meeting the eligibility criteria from the electronic databases explored. Table 2 shows the study characteristics of the 12 included studies in this systematic review and meta-analysis. Ten articles (83.3%) were published after 2010 with 100% of the studies published in the academic and peer-reviewed journal comprising of survey (n = 8), pilot study (n = 1), multifocal study (n = 1), prospective study (n = 1), and retrospective study (n = 1).

**Table 2:** The study characteristics of the 12 included studies EAP: Nonparticipant applicants; EP: Elective participants; GS: General surgery; MS: Medical school; NLI: Neuroanatomy lab initiative; NSIG: Neurological surgery interest group; SEAD: Surgical exploration and discovery; SIG: Student interest group.

Author ID	Study Design	Participants	Interventions	Outcomes
Lubelski et al. (2019) [22]	Online (electronic) survey	The United States' neurosurgery program directors comprising 110 programs with a median student body of 150 recruited in the study.	Surveying neurological surgery program directors to identify the number of medical students who underwent rotations and those matching into the residency of neurosurgery in their medical programs between 2010 and 2016.	Out of 110 directors, 30 provided complete responses. 52% of the medical institutions offered didactic neurological surgery training and lectures for first- and second-year medical programs. On the other hand, neurosurgical didacts were provided in the third- and fourth-year levels in 87% of the institutions. At the same time, the neurological surgery outreach program was the most common in integrating the students among 77% of the departments. In addition, other interventional forms and outreach methods constituted lecture series accounting for 57%, laboratory-based neurosurgery anatomy making up 40% and mentorship programs constituting 53%. Based on these respondents, about three students went through a rotation through a neurosurgical department, while one matched into neurological surgery every year.
Agarwal et al. (2013) [3]	A retrospectively divided multiphase initiative (a four-phased initiative) strategy	200 medical students from a single institution's experience went through the four phases of the initiative between 2002 and 2012. Eight students per rotation in Phase 1 totaled 48 students out of 200 in 2002.	A four-phase stepwise initiative designed to address and counter a deficit in the exposure of medical students to neurological surgery. Phase 1 (2002) encompassed a two-week rotation through the neurosurgical experience of the existing neurological clerkship (third-years). Phase 2 (2007) entailed active departmental recruitment of undergraduate medical students in a seven-year accelerated BS or MD programs and other preclinical students for a yearlong and summer research program. Phase 3 (2010) entailed inflexible clinical rotations done for a year. Under this level, more courses pertaining to neurosurgery were added, with third-year students having the opportunity to participate in a two-week elective, two- to four-week elective neurosurgery for fourth years as well as the elective based on endovascular neurosurgery. Finally, Phase 4, done in January 2012, involved approval of the neurosurgery interest group, which accelerated the improvement of neurological medical education through a synthesized didactic curriculum.	The implementation of the four-phased initiative dramatically increased the number of students matching into neurosurgery in the institution. The number of medical students in the summer program increased steadily, with four students enrolling in 2008, six in 2009, and eight students in 2010. Additionally, 10 medical students were enrolled in 2011, while the number increased to 12 in the 2012 summer program. In summary, with the effective execution of the initiative, the medical students at the institution got well conversant and acquainted with neurosurgical experience through the additional neurological surgery lectures, electives, and sessions. The formulation of neurosurgical interest groups elevated the interaction with the neurological surgery faculty.
Huq et al. (2020) [23]	Supplemental content survey	Five neurological surgery residents, together with a rotating faculty member, directed a total of 39 medical students, of which 51.5% were Caucasians and 21.2% represented traditionally underrepresented groups in medicine. In contrast, the remaining 36.3%, comprised of Asian medical students, underwent a hands-on operation of four specimens' retrosigmoid craniotomy.	The recruitment of medical students into neurological surgery using a focused neuroanatomy lab strategic initiative (NLI). The NLI was based on a cadaver lab event for the first-year medical students for gross anatomy.	Out of the 39-student cohort, 84.6% (33) completed the pre-event survey comprising 16 male and 17 female students. After the NLI and based on the responses from the survey, 54.5% chose a surgical specialty, while 30.3% had a surgical career as their top choice. Neurosurgery was the most common first choice with 18.2%, 15.2% for neurology, and 9.1% for interventional radiology. On the other hand, 18.2% of the students remained undecided on their specialty choice.
Kashkoush et al. (2017) [5]	Survey	University of Pittsburgh School of Medicine created the neurological surgery interest group (NSIG), comprising 100 student members with four appointed officers in October 2014. NSIG recruited 89 medical students comprising 33 female and 56 male students in the first year. Of the 89 students, 64 were first-year students, while 22 were second-year students, making up 97% of the membership. In the second year, 60 more members were recruited to the group. Additionally, a 14-medical students committee was formulated to conduct concerted study research in June 2015.	Creation and formulation of an NSIG to foster the interest of medical students' recruitment in neurosurgery. A total of 17 medical student-oriented programs were hosted and conducted by NSIG to augment students' interest in neurosurgery. The events aimed to provide medical students with acquittance in neurosurgery topics and didactic workshops to develop neuroradiology, skull management, concussion, and stimulation of the deep brain.	Based on the 14-student subset that conducted a resident-led research project, 35 manuscripts were produced by seven members of the committee, of which 19 were accepted as peer-reviewed journals. The significance of NSIG at the university led to increased students' matriculation into neurosurgery residencies. In 2014 alone, three medical students were matched into neurological surgery, while 2015 saw two others join the residence. Another two and five students were matched into neurosurgery in 2016 and 2017, respectively. From 2016 to 2017, three students of the seven were members of the established NSIG.
Zuccato and Kulkarni (2016) [24]	Survey	A total of 38 members of a surgical exploration and discovery (SEAD) initiative were recruited to participate in two program cycles.	The effectiveness of early surgical exposure of medical students on their interest in neurosurgery through the SEAD program.	97% (37) of the participants completed their survey. Before the intervention, 105 were uninterested in neurosurgery, while 25% were interested. However, after implementing the SEAD program, the interest increased from 10% to 38%, keeping the surgical specialty-associated lifestyle factors controlled. 81% of these students revealed that SEAD intervention had improved neurosurgical understanding. However, 62.2% showed that their exposure and experience with other surgical specialties reduced their interest in neurological surgery, while 21% considered SEAD program intervention had elevated their interests in pursuing neurosurgery. In addition, one student planned on organizing neurosurgical research, while 19% intended to explore neurosurgery further during their observational internships.
Grover et al. (2016) [25]	Multifocal approach study	The enrollment of students into student interest groups (SIG) increased significantly from 112 yearly (2008-2009) to 150 (2012-2013) membership to increase the interest of students in surgical specialties.	The multifocal approach entails recruiting students into SIG, where they are exposed to didactic presentations from the surgical faculty.	A parallel increase in the number of medical students matching into surgical specialties was observed. In the 2013 class alone, 37 students reached surgical specialty residencies, a record 85% increase over the past 10 years. Of the 37, seven (18.9%) students matched into neurological surgery in 2013, four of 22 (18.2%) students in 2008 significantly increased compared to 0% in 2004 and 8% in 2005.
Gawad et al. (2013) [26]	Pilot project study	A total of 20 medical students who had accomplished their first year of medical year school were recruited to participate in a SEAD program at the University of Toronto, Canada, to get acquainted with observational skills, hands-on workshop simulations, and informal surgical careers discussions. It was a requirement that all the participants fill out a questionnaire survey pertaining to previous surgical experience and demographic data.	Employing the SEAD program for early comprehensive surgical exposure in the medical school department of surgery. The participants underwent a two-week SEAD program comprising three main aspects: observerships, discussions, and workshops. In addition, they also attended a session entailing an Introduction to Surgery outlining the SEAD initiative, surgical equipment, preoperative and postoperative evaluation and care, and OR etiquette.	Out of the 20 participants, 19 (95%) students (first-year medical students) completed the SEAD program. The remaining student was excluded due to conflicting commitments. After successfully going through the SEAD program, 84.25% of the 19 students revealed an increased interest in surgical careers. However, the number of participants interested in neurosurgery prior to the SEAD program was four, with two ruling out pursuing neurosurgery with zero and 10 students interested in pursuing another surgical specialty after the intervention of the program.
Day et al. (2016) [27]	Likert-type survey	A total of 18 elective participants (EP) comprising fourth-year medical students of both years 2014 and 2015 who had qualified from the Alpert Medica School and had previously participated in a program based on the Introduction to Surgery were included in the survey during the fall of both 2013 and 2014. The survey was done based on an electronic (online) platform. All included 18 EPs managed through the Introduction to Surgery program before matching into surgical residency. This had the academic years of 2013 to 2014 and 2014 to 2015—the 18 EPs comprised seven first-year medical students, while the remaining 11 were second-year students. Besides, the outcomes of the 18-EPs cohort were compared against eight EPs that did not merit recruitment to participate in the program.	The effect of early medical school mentorship programs on the clerkship performance of medical students and their career choice after completing junior medical students. Introduction to Surgery program, semester-long, a resident-directed, preclinical elective initiative for the 18 EPs who were supposed to answer a Likert-type survey after that.	In the clerkship performance section, 50% of EPs were awarded honors, 37.5% and 31.5% for the nonparticipant applicants (EAs) and medical school class (MS), respectively. In the surgical career selection category, 55.6% of the EPs successfully matched into various surgical residencies compared to the 37.5% of the nonparticipant applicant group and 27.7% for the medical school class. Of the 18 EPs, two (11.1%) medical students selected GS compared to 3.7% and 25% of the MSs and EAs, respectively. In addition, 44.4% of EPs chose surgical specialty (SS) relatively compared to MSs and EAs selection corresponding to 24.1% and 13.5%, respectively.
Akhigbe and Sattar (2014) [28]	Prospective study entailing cross-sectional survey based on a Likert scale	A total of 60 medical students with an age bracket between 20 and 26 years with a corresponding median age of 23 years were recruited for the study. The 60-student cohort comprised 32 female and 28 male third-, fourth-,and fifth-year medical students from the Royal College of Surgeons of Ireland. All the recruited participants were unmarried, with 72% being non-Irish, while the remaining 28% (17) were citizens of Ireland.	The analysis of perceptions and attitudes of medical students toward neurological surgery in the Irish Royal College of Surgeons.	Out of the 60 students, 80% revealed that neurosurgery was inadequately taught. However, 78% still considered furthering their career in neurosurgery. Above 90% of the respondents indicated that neurological demanded long hours of operation, which was also associated with a long training period, with 88% stating that the Irish neurological surgery training was prolonged. However, 92% acknowledged the prestigious advantages of neurosurgery, although 87% revealed that neurosurgical specialty might impede family life.
Cloyd et al. (2008) [29]	Survey	A total of 73 first- and second-year medical students were recruited to participate in "Operating Room: Surgical Skills" in the winter of 2007 at the University of California, San Francisco Medical School. Each student participant was paired with a surgical mentor who majorly included a total of 24 surgeons from the general surgery division. A total of 59 first-year students comprising 31 female and 28 male medical students enrolled in the surgical skills elective. 61% (36) of 11 female and 25 male students out of 59 students decided to participate in the clinical experience elective.	The significance of surgical mentorship and experience gained from the operating room for pre-clerkship medical students. A total of 36 students were paired under the instruction of 24 surgeons in a three-month elective period in which they were to participate in at least two surgeries. After every surgery, the students, surgeons, and nurses in the operating room were required to fill out the questionnaires.	During the three-month elective period, 30 students participated in at least a procedure, while the remaining six did not makeup up the operating room.
Kozar et al. (2003) [30]	A survey using a Likert-type scale	210 first-year medical students from the University of Texas, Houston School of Medicine, were inquired for voluntary participation in a brief presentation in the surgery department.	The intervention of surgeons in influencing medical students in choosing their careers. The participants went through a preintervention, a one-hour intervention, and a postintervention survey followed by an informal Q&A group session. Of the 210 cohorts, 121 comprising 50 females and 66 males, with five more unidentified, completed the presurvey, whereas the remaining 45% (94) of the participants completed post-survey after attending the intervention.	Of the 121 students, 9% (11) acknowledged GS, while 24% selected surgical subspecialty as their first and main career choice. 15% of the 82 cohort changed their choice to GS, whereas the other group remained unchanged. Besides, GS was ranked by 28% in the post-survey and 26% in the presurvey as their second-based career choice.
Zuckerman et al. (2016) [31]	Survey through a Likert questionnaire	A total of 35 medical students comprising 17 first-years (four females and 13 males) and 18 second-year students (three females and 15 males) were enrolled over two years (2012 and 2013) in a survey conducted by Vanderbilt University School of Medicine.	Early exposure if neurological surgery electives to preclinical students boost their interest in neurosurgical residency. The extent of operative surgical exposure in which 100% (35) of students participated encompassed 135 operations (34 spine, 49 intracranial, 25 endovascular, 13 open vascular, and 14 functional procedures). 45% of the students attended a brain tumor clinic, while 25% attended a functional neurological surgery clinic.	At the end and the completion of the elective, the medical students were much more likely to consider neurological surgery as their career choice (p = 0.0001), had an insight of friendliness and collegial in attending physicians (p = 0.0002), perceived higher quality of life (p < 0.0001), and had a final belief of achieving the future neurosurgical career and lead a family (p = 0.001). However, the elective did not change the perception of students on difficulties associated with neurosurgical training.

Meta-analysis

Assessment of Risk of Bias

**Figure 2:**
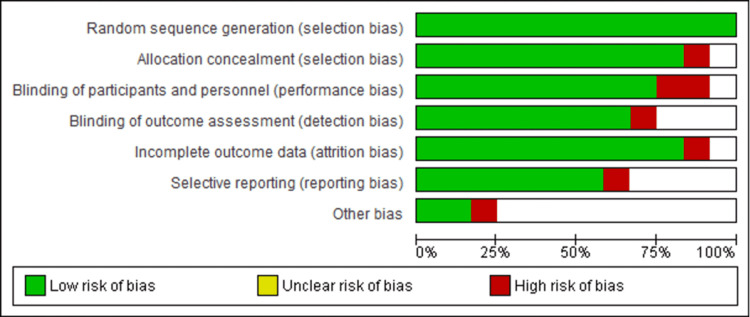
Risk of bias graph detailing the selection, performance, detection, attrition, reporting, and other bias of the 12 included studies on the improvement of the recruitment process into neurosurgery The red section implies that the outcomes of the studies can seriously implicate the confidence of the results, which led to ignoring articles of this nature. The green parts indicate low-risk studies, which are less likely to alter the outcomes. Thus, the 12 studies considered were of low risk. The unclear bias provides some doubts about the information that was ignored and excluded from the inclusion in the article.

In evaluating the risk of bias for the included studies, the items under consideration comprised attrition, reporting bias, detection, performance, and selection. Figure 2 shows the graph illustrating these items.

Risk of Bias Summary

**Figure 3:**
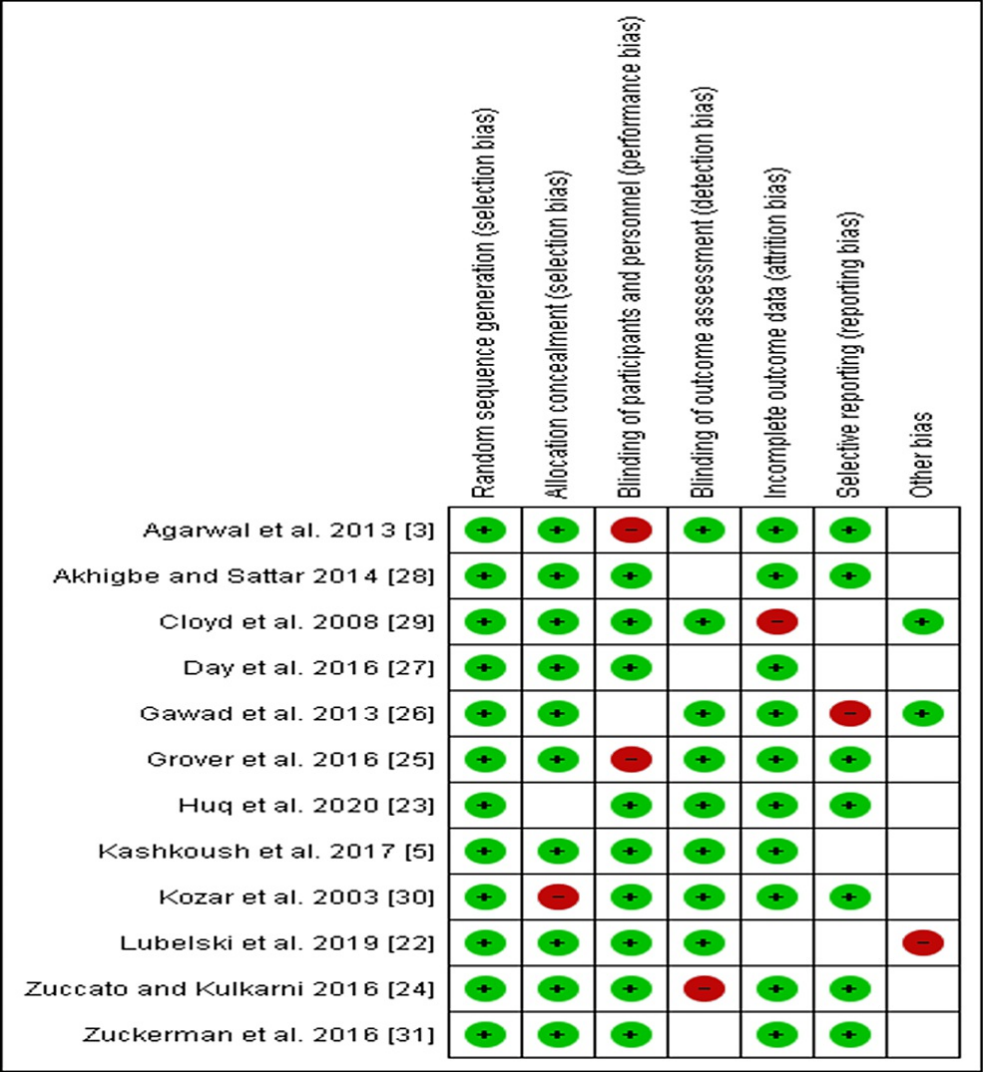
The summarized risk of bias of the various judgments made on the included studies and the overall interdependence of risks related to individual articles

The low risk in the included studies is indicated by a green circle shading (Figure 3). The red shading indicates high risk, while uncertain risks are marked by blank white space (Figure 3). The unclear risk implies insufficient data to make a clear judgment on various studies included in this current study.

Plots

Forest Plot

**Figure 4:**
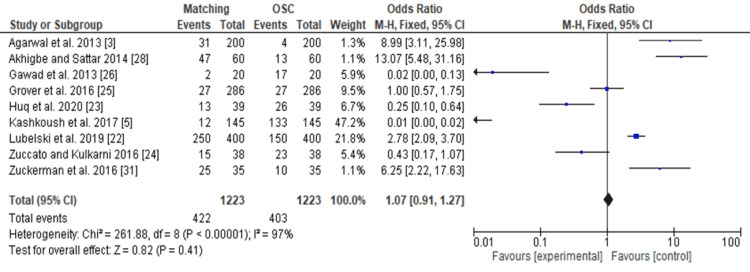
The forest plot showing the total number of students matching into neurological surgery residency after various interventional strategies of improving recruitment to neurosurgery

The forest plot (Figure 4) analyzes the total number of students matching into neurosurgery relative to those joining other surgical careers (OSC) and specialties. The forest analysis (Figure 4) data demonstrates that students who underwent interventional enrollment strategies are more likely to match into neurosurgery residencies (OR 1.07, 95% confidence interval [CI]: 0.91 to 1.27).

Funnel Plot

**Figure 5:**
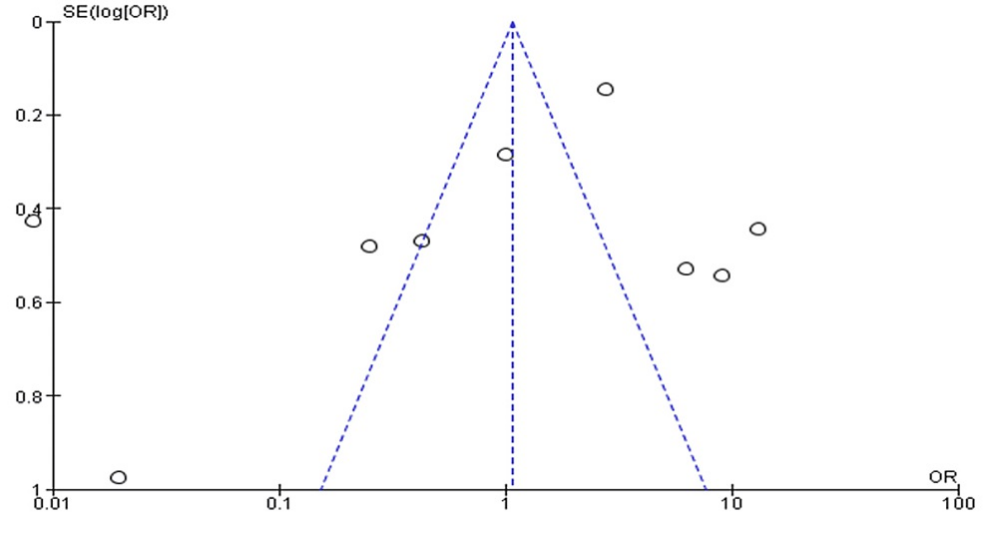
The funnel plot for the improvement of recruitment neurosurgery

The asymmetrical appearance of the funnel plot (Figure 5) implies the studies included in this study are heterogeneous as it can be demonstrated (Figure 5) that some studies are spread outside the 95% CI boundary.

Forest Plot

**Figure 6:**
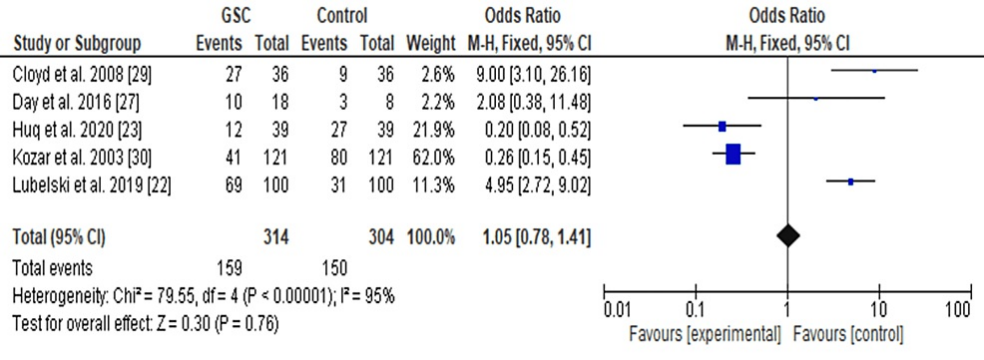
The forest plot of students who were interested and matched into surgical specialties including neurosurgery after going through various institutional programs

Further analysis with a forest plot (Figure 6) was also used to compare students based on interest in general surgery and other surgical specialties. The forest plot (Figure 6) indicates students matching in general surgical careers (GSC) and other surgical specialties relative to control (those who are totally uninterested). From this analysis, the forest plot demonstrates that students interested in neurosurgery are likely to match (OR 1.05, 95% CI: 0.78 to 1.41).

Funnel Plot

**Figure 7:**
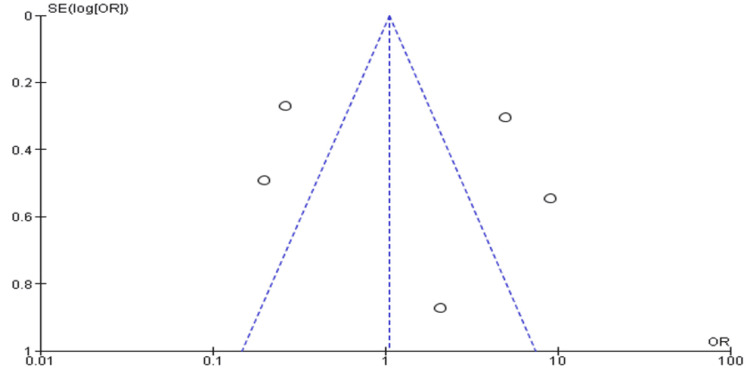
The funnel plot of students who were matched into surgical specialties including neurological surgery after undergoing various institutional programs

The funnel plot (Figure 7) is asymmetrical as most of the studies (indicated by small circles) are distributed outside the 95% confidence interval boundaries. The data set includes studies of students who matched into surgical specialties including neurosurgery after undergoing training through institutional programs.

Discussion

Summary of the Findings

The above results indicate that early exposure of medical students to neurosurgery significantly influences young clinicians' career choices in pursuing a surgical residency. The tabulated results show that numerous interventional programs such as SEAD, NLI, SIG, and NSIG adopted in various institutions aimed at increasing student acquaintance with neurosurgery were key to improving their interest in furthering their career in neurosurgery. Three of the included studies revealed that increased early exposure of the students to neurosurgery improves their interest in furthering their careers as neurosurgeons [24,31,3].

The funnel plots and forest plots generated from the outcomes of the included studies indicate the availability of bias and level of variations in study results between included studies. In Figure 4, all nine studies' 95% CIs mostly overlap. The p-value for heterogeneity (variations of study outcomes) of 0.41 and the I^2^ of 97% show that significant heterogeneity is detected. Consequently, it implies that the total number of students matching into neurosurgery residency after diverse interventional approaches to improving recruitment largely varied between the included studies. Similarly, in Figure 5, the 95% CIs of all the five studies largely overlap. The large p-value for heterogeneity of 0.76 and the I^2^ of 95% both indicate the detection of significant heterogeneity. It implies that interested students and those that matched into surgical specialties, including neurosurgery, largely differed after going through multiple institutional programs. On the other hand, both the funnel plots generated were asymmetrical. The asymmetrical plots are attributed to bias since all studies in open circles are of lower methodological quality. Consequently, they generated exaggerated intervention effect estimates on neurosurgery recruitment.

Main Findings Discussion

A number of previously conducted studies have cited that neuroscience is currently underrepresented in medical training and education despite being one of the cornerstones of a wide wing of clinical pathologies [17,32]. According to another study, the deficit in neurosurgical recruitment is exacerbated by the increasing aging population, resulting in high pressure demanding for neuromedical specialists. In addition, such a demographic change, along with the expected fall in the number of neurosurgery residencies by 2025, has made it imperative to assess the education, training, and recruitment of medical students to neurosurgery [33]. For this reason, studies have suggested a number of aspects needing reexamination in neuromedical education. Some of these key areas under consideration encompass the period and timing necessary for one to complete neurosurgery training, the existence and formulation of both formal and informal mentorship programs, and the availability and accessibility of third-year medical students to neurosurgery clerkships.

In light of this, another study indicated that the core clerkship period in neurosurgery training for third- and fourth-year medical students significantly impacted the number of students matching into neurosurgery. Moreover, this study revealed that there had been a significant increase in the number of recruitments for medical students into neurology for both foreign and the United States (US) medical graduates [34]. Besides, these findings are consistent and parallel with three of the included studies in this article. The three reports demonstrate that medical students participating in neurosurgery clerkship show greater interest and optimism in pursuing neurosurgical career residency [22,3,5]. In conjunction, however, one other study revealed that about 62% of surgical clerkship did not offer didactic lectures, with only 17% of medical institutions having a formal program for student mentorship. The mentorship program could be, in turn, utilized to accelerate neurosurgical research and exposure [6].

Additionally, the article revealed that insufficient neurosurgical clerkship could be articulated to the absence and lack of designated mentorship programs for the medical students. Concerning this, three of the included studies indicated that early mentorship programs for medical students played a significant role in influencing students to choose neurosurgery as their career path [27,29]. Early mentorship programs saw one student matched into neurological surgery yearly [22].

On the other hand, several studies have attributed the declining number of neurosurgeons to the difficulty of the training necessary for this specialty [35]. For instance, it was observed that a majority of medical students in the United States experienced neurosurgery as the most time-demanding and hence difficult specialty (p < 0.001) [36]. In conjunction, another survey in this article indicated that neurosurgery was inadequately taught, although most of these participants enrolled and chose neurosurgery as their career path [28]. In relation to this observation, several medical institutions have reevaluated and begun reconstruction of their medical school curriculum globally to counteract these hindrances to recruiting neurosurgical residence applicants. Therefore, programs have provided insights on the best alternatives to address both improving the students' interests and increasing the recruitment of medical students into neurosurgery residency. In this regard, some of the included studies outline efforts made by various institutions to increase the participation of medical students in neurosurgery. For instance, a four-step process was adopted in one study to increase early exposure and acquaintance of medical students with neurosurgery which consequentially led to an average of 3.8 student matches into neurological surgery per year [3].

Similarly, another previous study revealed efforts by a medical school to improve mentorship programs, teaching, and research in neurosurgery, which saw a significant increase in the number of students matching into neurosurgery (from 14 students between 2006 and 2010 to 30 between 2011 and 2014) with a p-value < 0.05 [37]. This study's finding collaborates with five articles that explored strategic programs employed across the world's medical institutions to help increase the number of neurosurgical resident applicants. Some of these strategies include the neuroanatomy lab initiative (NLI) program [23]; the creation of NSIG [5]; the student interest groups (SIG) program [25], which was associated with an 85% record of student matching into neurosurgery, and the surgical exploration and discovery (SEAD) program [24,26], which exhibited an increased interest of students in neurosurgery from 10% to 30% after its implementation. A significant number of medical students (82%) revealed that the SEAD program improved their understanding of neurological surgery [24]. However, in another study, the implementation of the SEAD program saw surgical students reconsider their special interests, with zero students showing interest in joining the neurosurgery resident despite initially being interested in neurology [26]. They shifted their interest to other surgical cases, while the initially interested members did not change significantly. Therefore, these programs' implementation offers future improvements and an increased number of aspiring medical students in neurosurgery residency and neuromedicine.

Assessment and Limitation of the Study

In the search process, only articles available in English were considered for inclusion, which signifies that the omission of studies published in other languages might have led to limited vital information on improving neurosurgery training recruitment. Similarly, studies encompassing other types of surgery were ignored, depriving this systematic review of vast data about increased recruitment into surgical residencies. Additionally, inconsistencies resulted in varying numbers of participants across the included studies, making it difficult to make a generalized conclusion on all studies. Nevertheless, most studies provided consistency in the number of students interested in neurosurgery residences. Thus, it was possible to correlate and harmonize results based on annual increments in the number of students absorbed into neurosurgery.

## Conclusions

Generally, several areas need to be prioritized and put into consideration in efforts to improve the recruitment of medical students to neurosurgical fields. It is worth noting that in many medical institutions' curricula, medical students may lack appropriate exposure to neuroscience in their first year of study. Such a factor often derails their interest in pursuing neurological surgery due to limited research experience. Therefore, medical educators, neuroscience specialists, and other key stakeholders need to consider reconstructing the neurosurgical curriculum to ease the prolonged study time and create and encourage structural programs to acquire medical students in neurosurgery.

First-year students need to be exposed to neurosurgery early enough to help and guide their ambitions and interests in becoming neurosurgeons in their future careers. In addition, based on the majority of the studies in this article, the medical institutions must develop structured programs such as the SEAD, NLI, and SIG programs aimed at acquainting medical students with enough knowledge to guide their interest through continued project involvement for neurological surgery productivity. Furthermore, some studies reveal that medical institutions must incorporate and provide continued mentorship programs for students during their medical studies. Quality mentorship is an essential integration to the neurological surgery training that is key in developing the professional development of the students, which in turn encourage entry into certain neuroscience specialty. Besides, including some senior students in the mentorship programs would certainly spike up the number of interested junior medical students equipping them with the necessary skills and knowledge required to motivate students in the course of their careers.
